# Integrated Transcriptomic Analysis Identifies ENO3 as a Candidate Lactate Metabolism‐Associated Biomarker in Colorectal Cancer Metastasis

**DOI:** 10.1002/cnr2.70723

**Published:** 2026-10-11

**Authors:** Jiahao Qian, Miaomiao Wang, Jiaqi Liu, Meiling Gao

**Affiliations:** ^1^ Central Research Laboratory, the First Affiliated Hospital of Soochow University Suzhou China; ^2^ Department of Dermatology The First Affiliated Hospital of Soochow University Suzhou China; ^3^ Department of Obstetrics and Gynecology The First Affiliated Hospital of Soochow University Suzhou Jiangsu China

**Keywords:** biomarker, colorectal cancer, ENO3, metabolic reprogramming, tumor metastasis

## Abstract

**Background and Objectives:**

Metastasis is the primary cause of mortality in colorectal cancer (CRC), a leading global malignancy. Although metabolic reprogramming is a hallmark of cancer, the stage‐associated transcriptional features of metabolic pathways during CRC progression remain incompletely understood. This study aimed to characterize transcriptionally inferred metabolic heterogeneity during CRC progression and identify candidate molecular factors associated with metastasis.

**Methods:**

We performed an integrated transcriptomic analysis using TCGA and GEO datasets and compared metabolic pathway signatures across normal tissues, primary tumors, and metastatic lesions. An exploratory tumor lactate metabolism score (TLMS) was developed using Cox regression and LASSO analysis. Consensus clustering identified metabolic subtypes, and their associations with computationally estimated immune‐cell signatures were assessed. The functional consequences of ENO3 loss were preliminarily evaluated using CRISPR/Cas9 knockout, cell proliferation assays, and an NSG tail‐vein experimental lung colonization model.

**Results:**

We observed a stage‐associated reversal‐like transcriptional pattern: primary tumors showed broad suppression of metabolic pathway signatures relative to normal tissues, whereas metastatic lesions showed reactivation of several signatures, including the pentose phosphate pathway. A six‐gene lactate metabolism‐related signature stratified patients into prognostic groups within the TCGA cohort. The high‐risk group showed higher ssGSEA‐inferred neutrophil abundance and enrichment of a neutrophil extracellular trap (NET)‐associated transcriptional signature. ENO3 expression was associated with poor outcome, while ENO3 knockout reduced MC38 cell proliferation and experimental lung colonization in NSG mice.

**Conclusions:**

CRC progression is accompanied by stage‐associated changes in transcriptomically inferred metabolic programs. TLMS and ENO3 represent exploratory candidate biomarkers that require independent clinical validation and further mechanistic investigation, particularly with respect to lactate metabolism and the tumor immune microenvironment.

## 1 Introduction

Colorectal cancer (CRC) remains one of the major causes of cancer‐related death worldwide, and metastatic dissemination, including liver and lung metastases as well as peritoneal metastasis (peritoneal carcinomatosis), is the main reason for poor patient survival [1, 2]. Despite advances in treatment, nearly half of patients with localized disease eventually develop metastasis, where the 5‐year survival rate declines from over 90% to approximately 12% [3, 4]. This therapeutic bottleneck underscores the need to characterize the biological processes associated with metastatic progression and identify candidate intervention strategies [5].

Metabolic reprogramming, a hallmark of cancer, has shifted our understanding of tumor biology beyond genetics [6]. The classic “Warburg effect” paradigm of persistent aerobic glycolysis is now being challenged [7]. A landmark study revealed that primary tumors can exhibit suppressed metabolism compared to normal tissues, whereas metastatic lesions are metabolically hyperactive—a phenomenon termed “metabolic reversal” [8]. This suggests a dynamic, stage‐specific metabolic plasticity is crucial for metastasis. However, the molecular switches governing this critical metabolic transition remain largely enigmatic.

A key consequence of this metabolic plasticity is the altered secretion of metabolites that can function as signaling molecules and reshape the tumor microenvironment (TME) [9, 10]. Lactate, once regarded as metabolic waste, is now recognized as an oncometabolite that may contribute to tumor progression and immune evasion [11, 12, 13]. By acidifying the TME, lactate can suppress cytotoxic T and NK cells [14, 15]. It has also been associated with changes in the myeloid compartment, including the recruitment and activation of tumor‐associated neutrophils, which can contribute to metastatic progression [16, 17, 18].

A lactate‐rich TME may provide conditions that support neutrophil‐associated metastatic processes, including neutrophil extracellular trap (NET) formation and protease secretion [19, 20]. Clinical and experimental studies have reported substantial neutrophil infiltration in CRC liver metastases and associations with metastatic outgrowth and treatment resistance [21, 22, 23]. However, the upstream tumor‐cell metabolic factors associated with these neutrophil‐related features remain incompletely understood. Defining these associations may help generate testable hypotheses concerning metabolic and immune interactions during CRC progression.

In this study, we investigated potential associations between lactate metabolism‐related transcriptional features, immune‐cell signatures, and CRC progression. Through integrated analysis of TCGA and GEO datasets, we observed a stage‐associated reversal‐like pattern in metabolic pathway signatures and identified a six‐gene lactate metabolism‐related signature comprising ENO3, TKTL1, ACADL, ACSL6, UGT2A3, and ALAD. The resulting exploratory TLMS was associated with patient outcome and ssGSEA‐inferred neutrophil abundance. ENO3 was subsequently selected for preliminary functional assessment, and its knockout reduced MC38 cell proliferation and experimental lung colonization in NSG mice. These findings nominate ENO3 as a candidate prognosis‐associated factor but do not establish a direct causal relationship among ENO3, lactate production, and neutrophil recruitment.

## 2 Materials and Methods

### 2.1 Acquisition and Preprocessing of Transcriptomic Data

Transcriptome profiles and matched clinical information for CRC samples were sourced from TCGA (TCGA‐COAD and TCGA‐READ projects, accessible at https://www.cancer.gov/ccg/research/genome‐sequencing/tcga). An additional microarray dataset (GSE41568) profiling primary versus metastatic CRC lesions was downloaded from GEO. Raw count matrices from TCGA were subjected to normalization via the DESeq2 R package (v1.50.2), whereas GEO data were analyzed using pre‐normalized expression values as deposited.

### 2.2 Identification of Differentially Expressed Genes and Pathway Enrichment

Genes exhibiting differential expression were detected using DESeq2, with selection criteria set at adjusted *p* < 0.05 and |log2FC| ≥ 1.5. To explore biological processes and signaling pathways associated with these genes, we performed gene set enrichment analysis (GSEA) on a ranked gene list ordered by log2 fold‐change values. Enrichment for Gene Ontology terms and KEGG pathways was evaluated using clusterProfiler (v4.18.2), with results deemed significant at FDR < 0.25.

### 2.3 Unsupervised Clustering for Molecular Subtype Discovery

Consensus clustering was applied to stratify samples into distinct molecular subtypes. The ConsensusClusterPlus package (v1.74.0) was employed with 1000 resampling iterations and an 80% subsampling ratio. The optimal number of clusters was selected based on cumulative distribution function (CDF) curves and consensus matrices.

### 2.4 Construction and Initial Evaluation of a Prognostic Signature

Metabolism‐related genes associated with patient survival were first screened via univariate Cox regression (survival package, v3.8.3), retaining those with *p* < 0.05. To refine the gene panel, least absolute shrinkage and selection operator (LASSO) regression with 10‐fold cross‐validation was performed using the glmnet package. The final prognostic model (TLMS) was built through multivariate Cox regression.

Survival stratification was visualized using Kaplan–Meier curves generated by the survminer package (v0.5.1), with patients divided into high‐ and low‐risk groups at the median score. Log‐rank tests were applied to assess statistical significance. Methodological details align with our prior work [24].

### 2.5 Estimation of Tumor‐Infiltrating Immune‐Cell Signatures

Immune‐cell composition within the TME was computationally estimated using single‐sample GSEA (ssGSEA) via the GSVA package (v2.4.4), covering 28 immune‐cell populations. Between‐cluster differences in the inferred immune‐cell scores were tested using Wilcoxon rank‐sum tests, with Benjamini–Hochberg correction applied for multiple testing. These ssGSEA scores represent transcriptome‐based estimates and were not interpreted as direct histological measurements of immune‐cell abundance.

### 2.6 Cell Lines and Reagents

MC38 murine colon adenocarcinoma cells and HEK293T human embryonic kidney cells were obtained from ATCC. Cells were propagated in DMEM (Gibco) containing 10% FBS (Gibco) and 1% penicillin–streptomycin (Gibco) and incubated at 37°C under 5% CO_2_.

### 2.7 Generation of ENO3 Knockout Cell Lines

To ablate ENO3 expression in MC38 cells, a guide RNA (5′‐CTGCAAGATCTGCGATGTGT‐3′) targeting murine Eno3 was inserted into lentiCRISPRv2 (Addgene #52961). Lentiviral particles were generated in HEK293T cells via co‐transfection of the sgRNA construct with psPAX2 (#12260) and pMD2.G (#12259) using Lipofectamine 3000 (Invitrogen #L3000015). Culture supernatants were collected 48 h later, passed through 0.45‐μm filters (Millipore #SLHV033RB), and applied to MC38 cells. Transduced populations were enriched by puromycin selection (5 μg/mL, InvivoGen #ant‐pr‐1) over 7 days. Monoclonal lines were isolated by fluorescence‐activated cell sorting (BD FACSAria III), and loss of ENO3 protein was verified by immunoblotting.

### 2.8 Immunoblot Analysis

Whole‐cell lysates were prepared in RIPA buffer (Beyotime #P0013C) supplemented with protease inhibitors. Protein quantification was performed using a BCA assay kit (Vazyme #E112‐01). Samples (30 μg per lane) were resolved by SDS‐PAGE, transferred onto PVDF membranes (Vazyme #E801‐01), and blocked with 5% non‐fat milk in TBST for 1 h at room temperature. Membranes were probed overnight at 4°C with antibodies against ENO3 (1:1000, Invitrogen #MA563785) and GAPDH (1:3000, CST #2118S), followed by 1‐h incubation with HRP‐conjugated secondary antibodies (1:5000, CST #7074S). Chemiluminescent signals were developed using ECL substrate (Vazyme #E423‐01) and captured on a Bio‐Rad ChemiDoc imaging system.

### 2.9 Cell Proliferation Assay

Proliferation rates were quantified using CCK‐8 reagent (Vazyme #A311‐01). WT and ENO3‐KO MC38 cells were plated at 2000 cells per well in 96‐well plates. At 24, 48, 72, and 96 h, 10 μL of CCK‐8 solution was added to each well and incubated for 3 h at 37°C in the dark. Optical density at 450 nm was recorded using a microplate reader. Each condition was tested in triplicate across three independent experiments.

### 2.10 Animal Experiments

All procedures involving animals were conducted under protocols approved by the Animal Ethics Committee of Soochow University (Approval #202504A0043). Female NSG mice (6–8 weeks old, Beijing Vital River) were maintained under specific pathogen‐free conditions and randomly allocated into two cohorts (*n* = 5 each). The NSG model was selected to provide a preliminary assessment of the tumor‐cell‐intrinsic effect of ENO3 loss on experimental lung colonization in an immunodeficient background and was not designed to evaluate an intact tumor immune microenvironment. MC38 WT or ENO3‐KO cells (5 × 10^5^) in 100 μL PBS were administered by tail‐vein injection. Mice were sacrificed on day 20, and the lungs were excised, fixed in 4% paraformaldehyde, and processed for paraffin embedding. Experimental lung colonization burden was quantified by counting surface pulmonary nodules in a blinded manner. Histological evaluation was performed using H&E staining (FuncBio Biotechnology, Suzhou).

### 2.11 Statistics

Experimental data were analyzed using GraphPad Prism 9.0. Comparisons between two groups were conducted using two‐tailed Wilcoxon rank‐sum tests (nonparametric) or unpaired *t*‐tests (parametric), as appropriate. Longitudinal data involving multiple groups were evaluated by two‐way ANOVA with Sidak's post hoc test. Bioinformatic analyses were executed in R 4.5.2. Results are expressed as mean ± SD, with *p* < 0.05 indicating statistical significance.

## 3 Results

### 3.1 Transcriptomic Metabolic Pathway Signatures Show Stage‐Associated Patterns During CRC Progression

To characterize transcriptomic features of metabolic pathways during CRC progression, we first compared primary tumors and adjacent normal tissues in the TCGA cohort. GSEA indicated broad suppression of several metabolic pathway signatures in primary tumors, including signatures related to the tricarboxylic acid cycle, lipid metabolism, and amino acid metabolism (Figure 1A). In contrast, comparison of primary and metastatic samples in the GSE41568 dataset showed relative reactivation of several metabolic pathway signatures in metastatic lesions (Figure 1B). Glycolysis and the pentose phosphate pathway were among the positively enriched pathways in metastatic samples (Figure 1C,D). These data suggest a stage‐associated reversal‐like pattern at the transcriptional level; they do not directly measure metabolic activity or flux.

**FIGURE 1:**
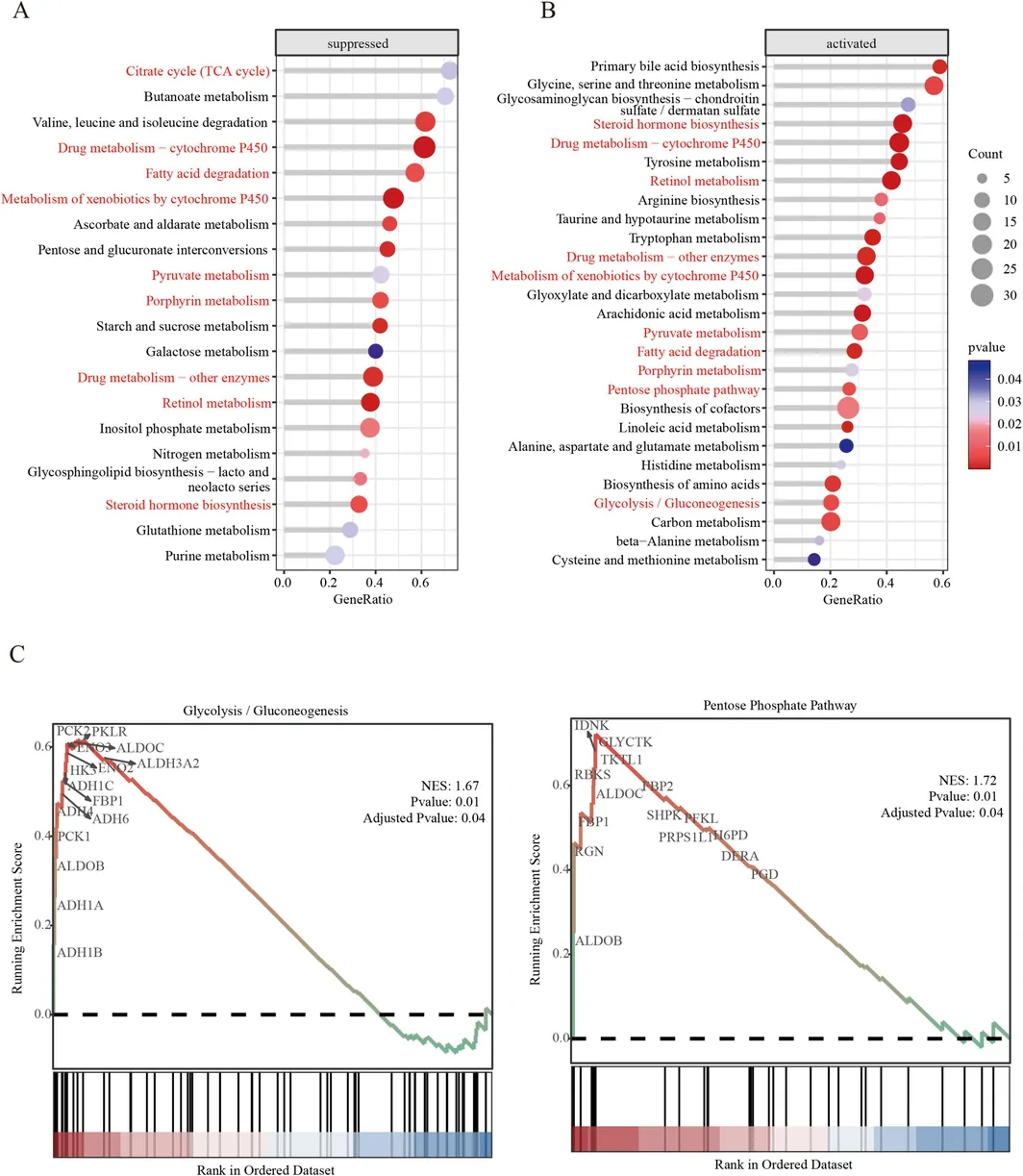
Transcriptomic metabolic pathway signatures show stage‐associated patterns during CRC progression. (A) Gene set enrichment analysis (GSEA) of metabolic pathways comparing colorectal cancer (CRC) tumor samples to normal adjacent tissues. The dot plot displays metabolic pathways significantly negatively enriched in tumor samples, with their normalized enrichment score (NES) and false discovery rate (FDR) indicated. (B) GSEA of metabolic pathways comparing metastatic CRC samples to primary tumor samples. The dot plot displays metabolic pathways significantly positively enriched in metastatic samples, with their NES and FDR indicated. (C) Representative GSEA enrichment plots demonstrating the positive enrichment of the “Glycolysis/Gluconeogenesis” pathway (left) and the “Pentose Phosphate Pathway” (PPP) (right) in metastatic CRC samples compared to primary tumors. Each plot displays the enrichment score curve, the ranked list metric, and the positions of core enrichment genes.

### 3.2 Lactate Metabolism‐Related Molecular Subtypes Are Associated With Inferred Neutrophil Abundance and Prognosis

Given the association between metastasis and poor clinical outcomes, we used univariate Cox regression combined with LASSO analysis to screen for prognosis‐associated candidate genes within key metabolic pathways. This analysis identified six lactate metabolism‐related genes associated with patient survival: ENO3, TKTL1, ACADL, ACSL6, UGT2A3, and ALAD (Figures 2A and S1A).

**FIGURE 2:**
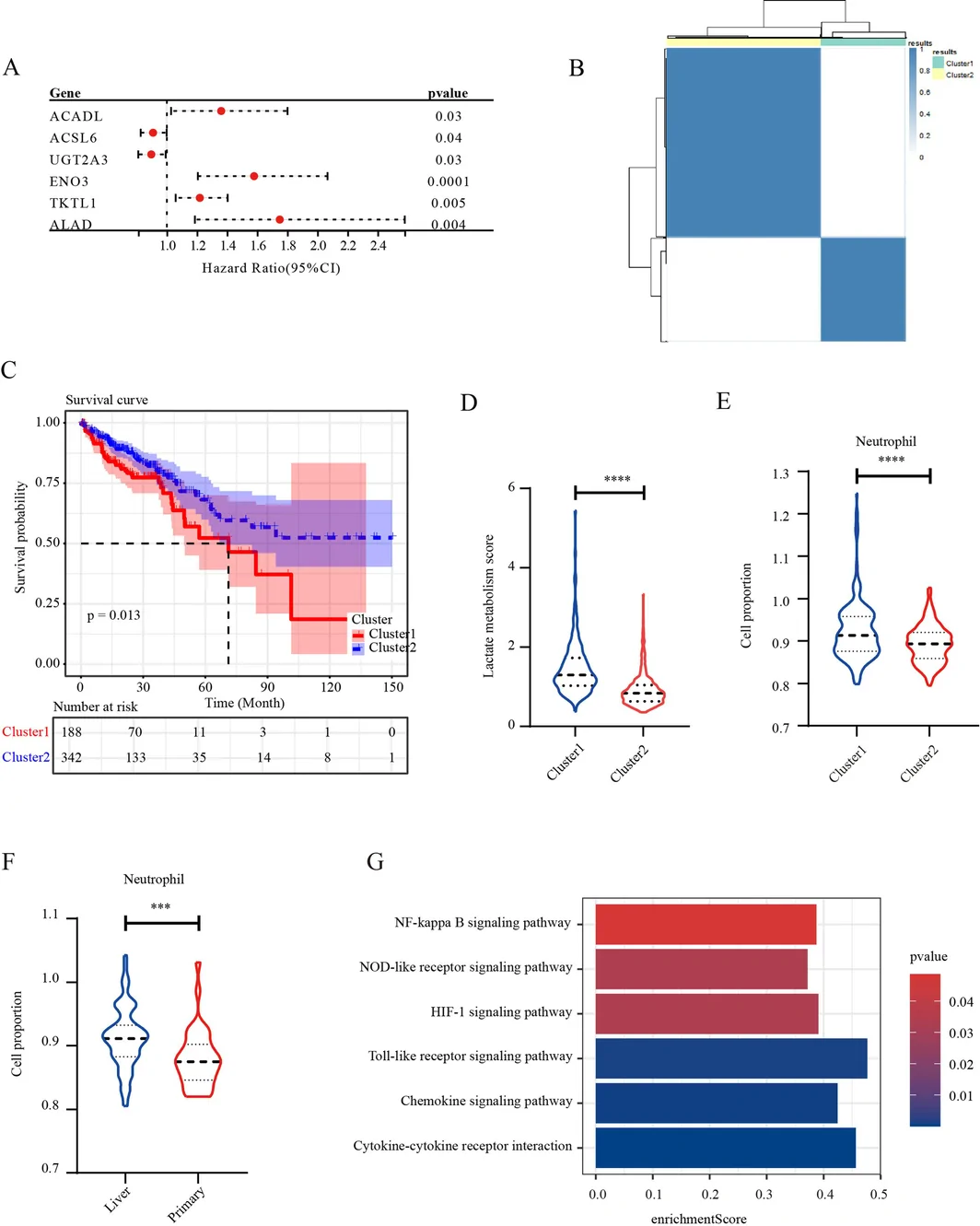
Lactate metabolism‐related molecular subtypes are associated with inferred neutrophil abundance and prognosis. (A) Forest plot of univariate Cox proportional hazards regression analysis for lactate metabolism‐related genes. The plot displays the hazard ratios (HRs) and their 95% confidence intervals (CIs) for overall survival (OS). Six genes significantly associated with prognosis are highlighted. (B) Consensus clustering heatmap based on the expression of the six prognostic lactate metabolism‐related genes. The heatmap visualizes two distinct molecular subtypes, designated as Cluster 1 and Cluster 2. (C) Kaplan–Meier survival analysis of OS for patients stratified by the two molecular subtypes (Cluster 1 vs. Cluster 2). The *p*‐value was calculated using the log‐rank test. (D) Violin plot comparing the lactate metabolism risk scores between Cluster 1 and Cluster 2. The risk score for each patient was calculated using a formula derived from the multivariate Cox regression model. (E) Violin plot comparing ssGSEA‐inferred neutrophil scores between Cluster 1 and Cluster 2. (F) Violin plot comparing ssGSEA‐inferred neutrophil scores between primary and liver metastatic tumor samples. Statistical significance in (D–F) was determined by a two‐tailed Wilcoxon rank‐sum test. *p < 0.05, **p < 0.01, ***p < 0.001, ****p < 0.0001. (G) Bar plot of KEGG pathway enrichment analysis for differentially expressed genes (DEGs) between Cluster 1 and Cluster 2. Bars represent the top pathways enriched in Cluster 2, ranked by −log10(FDR).

Based on the expression of these six genes, consensus clustering of the TCGA cohort stratified patients into two molecular subtypes, Cluster 1 and Cluster 2 (Figure 2B). Patients in Cluster 1 exhibited shorter overall survival (Figure 2C). We subsequently developed an exploratory quantitative TLMS, and higher scores were associated with the poor‐prognosis Cluster 1 within the TCGA cohort (Figures 2D and S1B). Because the signature has not been validated in an independent prognostic cohort, its clinical applicability remains to be established.

We next examined transcriptomic features associated with the poor prognosis of Cluster 1. ssGSEA showed a higher computationally inferred neutrophil score in Cluster 1 (Figure 2E). A similar pattern was observed when primary tumors and liver metastatic samples were compared, with higher inferred neutrophil scores in the metastatic group (Figure 2F). Pathway analysis of differentially expressed genes between the clusters showed enrichment of pro‐inflammatory and cytokine‐related pathways, together with PD‐1/PD‐L1 checkpoint‐related and NET‐associated gene signatures (Figures 2G and S1C). These results indicate associations among the lactate metabolism‐related signature, prognosis, and neutrophil‐related transcriptional features; they do not constitute direct evidence of neutrophil infiltration or NET formation.

### 3.3 ENO3 Is Associated With CRC Prognosis and Contributes to Tumor‐Cell Phenotypes

To prioritize candidate genes associated with CRC progression, we examined the six‐gene signature in relation to expression and prognosis. ENO3 was upregulated in CRC tumors compared with normal tissues (Figure 3A), and higher ENO3 expression was associated with shorter overall survival (Figure 3B). These analyses identify ENO3 as a prognosis‐associated candidate but do not establish its independent prognostic value.

**FIGURE 3:**
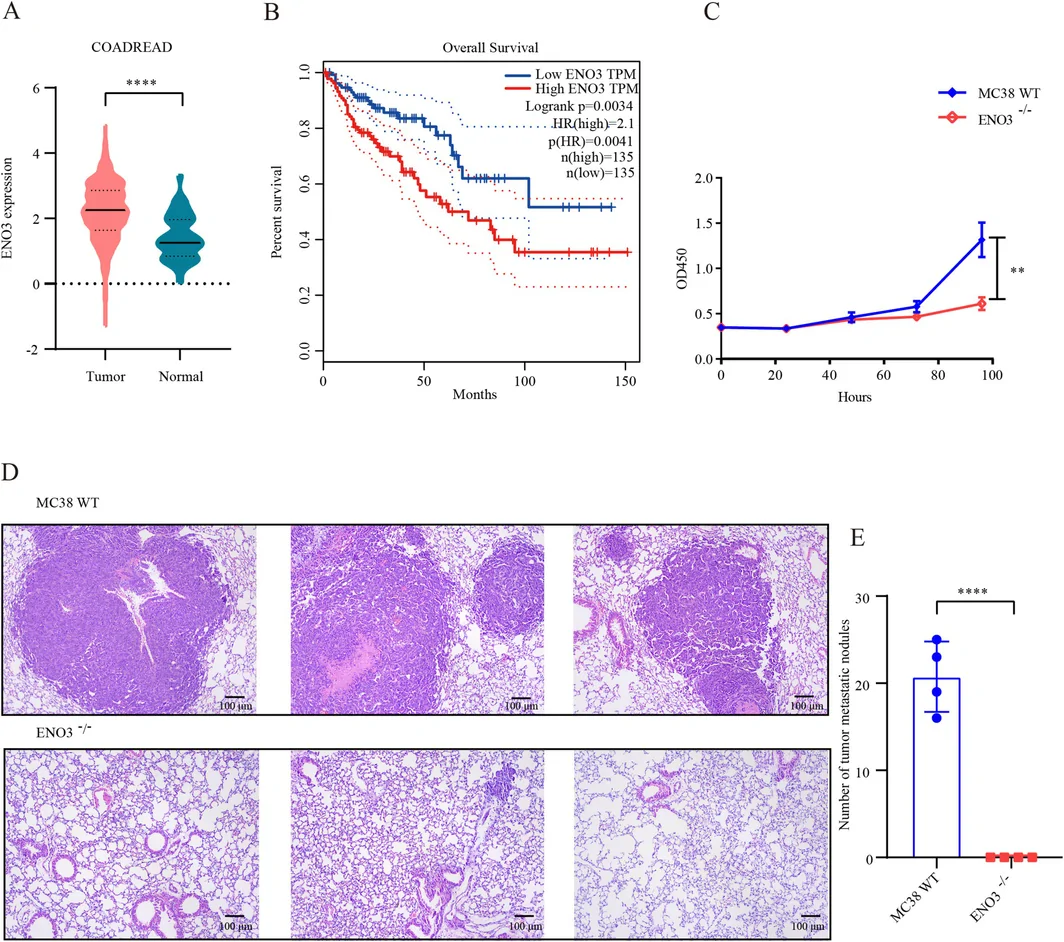
ENO3 is associated with CRC prognosis and contributes to tumor‐cell phenotypes. (A) Violin plot showing the differential expression of ENO3 in CRC tumor tissues compared to adjacent normal tissues, utilizing data from the TCGA COADREAD cohort. Statistical significance was determined by a two‐tailed Wilcoxon rank‐sum test. *p < 0.05, **p < 0.01, ***p < 0.001, ****p < 0.0001. (B) Kaplan–Meier survival curve illustrating the OS of CRC patients stratified by high and low ENO3 expression levels (grouped by the median expression value). The *p*‐value was calculated using the log‐rank test. (C) CCK‐8 cell proliferation curve comparing the viability of MC38 wild‐type (WT) and ENO3 knockout (KO) cells over a period of 96 h. Data are presented as mean ± SD from *n* = 3 biological replicates. Statistical analysis was performed using two‐way ANOVA with Sidak's multiple comparisons test. *p < 0.05, **p < 0.01, ***p < 0.001, ****p < 0.0001. (D) Hematoxylin and eosin (H&E) staining of lung tissues from NSG mice inoculated by tail‐vein injection with MC38 WT or ENO3‐KO cells, showing pulmonary lesions in the experimental lung colonization model. Representative images are shown at ×100 magnification. Scale bar, 100 μm. (E) Quantification of pulmonary tumor nodules in NSG mice inoculated with MC38 WT or ENO3‐KO cells. Data are presented as mean ± SD. Each dot represents an individual mouse (*n* = 5 mice per group). Statistical significance was determined using a two‐tailed unpaired *t*‐test.

To preliminarily assess the functional contribution of ENO3, we generated ENO3‐KO MC38 cell lines using CRISPR/Cas9 technology (Figure S2). CCK‐8 assays showed that ENO3 loss reduced MC38 cell proliferation (Figure 3C). In the NSG tail‐vein experimental lung colonization model, mice injected with ENO3‐KO MC38 cells developed a lower pulmonary lesion burden than mice injected with WT cells (Figure 3D,E). These findings support a potential tumor‐cell‐intrinsic contribution of ENO3 to proliferation and experimental lung colonization. Because the experiment was performed in immunodeficient NSG mice, it was not used to infer ENO3‐dependent regulation of the intact tumor immune microenvironment.

Taken together, the transcriptomic analyses and preliminary functional experiments identify ENO3 as a candidate factor associated with CRC progression. However, the present data do not directly demonstrate that ENO3 regulates lactate production, chemokine secretion, neutrophil recruitment, or the broader stage‐associated metabolic transcriptional pattern.

## 4 Discussion

Our analysis identified a stage‐associated transcriptional pattern in CRC: primary tumors showed lower enrichment of multiple metabolic pathway signatures than adjacent normal tissues, whereas metastatic samples showed relative reactivation of several signatures, including the pentose phosphate pathway. A related difference between primary solid tumors and metastases has previously been reported by Bartman et al. [8]. The present study extends this concept to transcriptomic datasets in CRC. Because pathway enrichment does not directly measure metabolite abundance or metabolic flux, these findings should be interpreted as transcriptionally inferred metabolic programs rather than direct evidence of metabolic activity.

This reversal‐like transcriptional pattern may reflect adaptation to distinct microenvironmental pressures. At the primary site, reduced expression of selected metabolic pathways may be associated with adaptation to nutrient limitation and immune pressure. In contrast, metastatic tumor cells face circulatory stress, distant‐organ colonization, and microenvironmental reconstruction, processes that may favor increased expression of metabolic programs [25]. Enrichment of the pentose phosphate pathway is potentially relevant because this pathway supplies NADPH for redox defense and ribose phosphate for nucleotide synthesis [26]. These biological interpretations remain hypotheses that require direct metabolic validation.

ENO3 emerged from the six‐gene signature as a prognosis‐associated candidate. ENO3 has traditionally been associated with muscle energy metabolism [27, 28], while previous work has also implicated it in CRC cell glycolysis and progression [28]. In the present study, ENO3 was upregulated in CRC and associated with poor outcome, and its knockout reduced MC38 proliferation and experimental lung colonization. These findings support a functional contribution of ENO3 to tumor‐cell phenotypes but do not establish ENO3 as a central orchestrator of metastasis or demonstrate that its effects are mediated by lactate metabolism.

Another observation was the association between the lactate metabolism‐related signature and neutrophil‐related transcriptional features. High TLMS scores were associated with higher ssGSEA‐inferred neutrophil scores, consistent with the reported pro‐metastatic roles of neutrophils in CRC [21, 29]. Nevertheless, transcriptomic deconvolution does not directly quantify neutrophil infiltration, and enrichment of a NET‐associated gene set does not demonstrate NET formation. Existing literature provides plausible routes through which lactate, inflammatory cytokines, and chemokines may influence neutrophil behavior, but the present data do not show that ENO3 alters lactate production, chemokine secretion, or neutrophil recruitment. Direct testing will require a dedicated mechanistic study using immune‐competent models, metabolic measurements, immune profiling, and tumor cell‐neutrophil interaction assays.

The enrichment of the PD‐1/PD‐L1 checkpoint‐related gene set further suggests that the high‐risk subgroup may exhibit an immunoregulatory transcriptional phenotype. Durvalumab, a PD‐L1‐blocking antibody, has been evaluated with tremelimumab in refractory CRC and showed an exploratory overall‐survival signal in the Phase II CO.26 study [30]. However, our pathway enrichment results do not demonstrate PD‐L1 protein expression or establish TLMS as a predictive biomarker for PD‐L1 blockade. Validation in treatment‐defined cohorts incorporating mismatch‐repair status, tumor mutational burden, and direct PD‐L1 assessment would be necessary before any therapeutic inference can be made.

### 4.1 Clinical Translation Value and Therapeutic Implications

The six‐gene signature and TLMS provide an exploratory framework for transcriptomic risk stratification. Within the TCGA cohort, higher TLMS values were associated with poorer outcome and distinct immune‐related transcriptional features. However, the model was developed and initially evaluated in the same cohort and has not undergone independent prognostic validation or comprehensive adjustment for clinical and molecular variables. TLMS should therefore be regarded as a candidate research signature rather than a clinically applicable decision tool.

### 4.2 Study Limitations and Future Perspectives

Several limitations should be acknowledged. First, the relationship between ENO3 and lactate metabolism was inferred from transcriptional signatures; lactate production, glucose consumption, and metabolic flux were not directly measured. Second, neutrophil abundance and NET‐related activity were inferred computationally and were not validated by histological, flow‐cytometric, or functional assays. Bulk transcriptomic data also do not allow definitive assignment of ENO3 expression to tumor, stromal, or immune‐cell populations. Although the MC38 experiments confirm ENO3 expression and functional relevance in tumor cells, they do not exclude expression in other components of the TME.

Third, the in vivo experiment used an immunodeficient NSG tail‐vein model. This system provides preliminary information about tumor‐cell survival and lung colonization but bypasses earlier steps of the metastatic cascade, including local invasion and intravasation, and cannot determine whether ENO3 regulates neutrophil recruitment or an intact antitumor immune response. Fourth, the TLMS was not validated in an independent prognostic cohort, and the independent prognostic value of ENO3 was not assessed using a comprehensive multivariable model incorporating TNM stage and molecular characteristics. Consequently, the clinical generalizability of the signature remains uncertain.

Future work should combine direct metabolic measurements with immune‐competent syngeneic or orthotopic CRC models, neutrophil profiling, and single‐cell or spatial approaches to resolve cell‐type‐specific ENO3 expression and potential tumor‐immune interactions. Artificial intelligence and machine‐learning approaches may also facilitate future integration of transcriptomic, pathological, spatial, and clinical information for refined risk stratification [31]. However, such approaches require large multicenter datasets, external validation, interpretability, and careful control of algorithmic bias.

## 5 Conclusions

In summary, this study identifies stage‐associated changes in transcriptomically inferred metabolic programs during CRC progression and nominates ENO3 as a prognosis‐associated candidate factor. ENO3 knockout reduced MC38 proliferation and experimental lung colonization in immunodeficient NSG mice, providing preliminary evidence of a tumor‐cell‐intrinsic functional contribution. The observed associations among TLMS, neutrophil‐related transcriptional features, and outcome are hypothesis‐generating and do not establish a causal lactate‐neutrophil mechanism. Independent clinical validation and dedicated metabolic and immune studies will be required to define the biological and translational significance of these findings.

## Author Contributions

**Jiahao Qian:** methodology, validation, visualization, software. **Miaomiao Wang:** methodology, software, validation, visualization. **Jiaqi Liu:** funding acquisition, writing – review and editing, project administration, supervision, resources. **Meiling Gao:** writing – original draft, writing – review and editing, conceptualization, investigation, funding acquisition, methodology, formal analysis, project administration, supervision, visualization, resources, data curation.

## Funding

This work was supported by the Natural Science Foundation of the First Affiliated Hospital of Soochow University (BXQN2024038) and the Suzhou Youth Fund for Basic Research Projects (SSD2025072). The article processing charge was supported by the Natural Science Foundation of the First Affiliated Hospital of Soochow University (BXQN2025010). Meiling Gao is supported by the Suzhou Gusu Health Talents Project.

## Ethics Statement

All animal experiments were conducted in strict accordance with protocols reviewed and approved by the Institutional Animal Care and Use Committee (IACUC) of Soochow University (Approval No.: 202504A0043). All procedures adhered to the guidelines outlined in the National Institutes of Health's Guide for the Care and Use of Laboratory Animals (8th Edition).

## Consent

The authors have nothing to report.

## Conflicts of Interest

The authors declare no conflicts of interest.

## Supporting information


**Figure S1:** Development and validation of a lactate metabolism‐related prognostic signature in CRC.
**Figure S2:** Validation of ENO3 knockout in MC38 cells.

## Data Availability

All data supporting our conclusions are included in the manuscript and Supplementary Materials.
