# Supplementary material for: Integrated Transcriptomic Analysis Identifies ENO3 as a Candidate Lactate Metabolism‐Associated Biomarker in Colorectal Cancer Metastasis
Source: Cancer Rep (Hoboken). 2026 Oct 11;9(10):e70723. doi: 10.1002/cnr2.70723 (PMC13655375; doi:10.1002/cnr2.70723)
Supplement: Supplementary file 1 [file CNR2-9-e70723-s001.docx]

**Supplementary Materials for**

**Integrated transcriptomic analysis identifies ENO3 as a candidate lactate metabolism-associated biomarker in colorectal cancer metastasis**

**Running title:** ENO3 as a candidate biomarker in CRC metastasis

Jiahao Qian^1#^, Miaomiao Wang^2#^, Jiaqi Liu^3*^, Meiling Gao^1*^

**Affiliations:**

^1^ Central research laboratory, The First Affiliated Hospital of Soochow University, Suzhou, 215006, China.

^2^ Department of Dermatology, The First Affiliated Hospital of Soochow University, Suzhou, 215006, China.

^3^ Department of Obstetrics and Gynecology, The First Affiliated Hospital of Soochow University, Suzhou, Jiangsu, 215006, China.

These authors contributed equally: Jiahao Qian and Miaomiao Wang.

*Corresponding authors: Meiling Gao (meili_G@126.com) and Jiaqi Liu (jiaqi__liu@163.com).

**Corresponding authors**

**Professor, Meiling Gao, Ph.D.,**

The First Affiliated Hospital of Soochow University

Central Research Laboratory

Shizi Road 188, 215006 Suzhou, China

Email: [meili_G@126.com](mailto:meili_G@126.com)

**Jiaqi Liu**

The First Affiliated Hospital of Soochow University

Department of Obstetrics and Gynecology

Suzhou, Jiangsu, 215006, China

Email: jiaqi__liu@163.com

This file includes:

Figure S1 and S2

**Figure S1. Development and validation of a lactate metabolism-related prognostic signature in CRC.**

(A) LASSO Cox regression analysis used to select prognostic genes from the initial candidates identified by univariate Cox regression. The plot shows the coefficient profiles of the candidate genes as the regularization parameter (lambda) varies. The vertical dashed line indicates the optimal lambda value chosen, resulting in the selection of a six-gene signature.

(B) Kaplan-Meier survival analysis comparing the OS of patients stratified into high-risk and low-risk groups. Patient stratification was based on the median value of the lactate metabolism risk score, which was calculated using the multivariate Cox model. Statistical significance was determined by the log-rank test.

(C) GSEA of pathways in Cluster 1 versus Cluster 2. The PD-1 checkpoint pathway (left) and the Neutrophil Extracellular Trap Formation pathway (right) were significantly enriched in Cluster 1.

**Figure S2. Validation of ENO3 knockout in MC38 cells.**

Western blot analysis confirming the knockout efficiency of ENO3 in MC38 cells. Cell lysates from wild-type (WT) and ENO3 knockout (KO) MC38 cells were subjected to Western blot analysis using antibodies against ENO3 and GAPDH. GAPDH served as the loading control. The results demonstrate complete absence of ENO3 protein expression in ENO3 KO cells compared to WT controls, confirming successful gene knockout.
